# The Crz1/Sp1 Transcription Factor of *Cryptococcus neoformans* Is Activated by Calcineurin and Regulates Cell Wall Integrity

**DOI:** 10.1371/journal.pone.0051403

**Published:** 2012-12-12

**Authors:** Sophie Lev, Desmarini Desmarini, Methee Chayakulkeeree, Tania C. Sorrell, Julianne T. Djordjevic

**Affiliations:** 1 Centre for Infectious Diseases and Microbiology, Sydney Medical School and Westmead Millennium Institute, University of Sydney, Westmead, New South Wales, Australia; 2 Faculty of Medicine, Siriraj Hospital, Mahidol University, Bangkok, Thailand; Dartmouth, United States of America

## Abstract

*Cryptococcus neoformans* survives host temperature and regulates cell wall integrity via a calcium-dependent phosphatase, calcineurin. However, downstream effectors of *C. neoformans* calcineurin are largely unknown. In *S. cerevisiae* and other fungal species, a calcineurin-dependent transcription factor Crz1, translocates to nuclei upon activation and triggers expression of target genes. We now show that the *C. neoformans* Crz1 ortholog (Crz1/Sp1), previously identified as a protein kinase C target during starvation, is a *bona fide* target of calcineurin under non-starvation conditions, during cell wall stress and growth at high temperature. Both the calcineurin-defective mutant, Δ*cna1,* and a *CRZ1/SP1* mutant (Δ*crz1*) were susceptible to cell wall perturbing agents. Furthermore, expression of the chitin synthase encoding gene, *CHS6,* was reduced in both mutants. We tracked the subcellular localization of Crz1-GFP in WT *C. neoformans* and *Δcna1* in response to different stimuli, in the presence and absence of the calcineurin inhibitor, FK506. Exposure to elevated temperature (30–37°C vs 25°C) and extracellular calcium caused calcineurin-dependent nuclear accumulation of Crz1-GFP. Unexpectedly, 1M salt and heat shock triggered calcineurin-independent Crz1-GFP sequestration within cytosolic and nuclear puncta. To our knowledge, punctate cytosolic distribution, as opposed to nuclear targeting, is a unique feature of *C. neoformans* Crz1. We conclude that Crz1 is selectively activated by calcium/calcineurin-dependent and independent signals depending on the environmental conditions.

## Introduction

*Cryptococcus neoformans var. grubii* causes life-threatening meningo-encephalitis especially in immunosuppressed individuals, including recipients of organ transplants. In this group, mortality approaches 40% in those with central nervous system infection [1], [2]. To prevent rejection of the transplanted organ, patients are treated with calcineurin inhibitor-based immunosuppressants that block signal transduction events required for T-cell activation.

Calcineurin is a conserved Ca^2+^-calmodulin-dependent serine-threonine-specific protein phosphatase consisting of a catalytic (CNA) and regulatory (CNB) subunit. The structure of calcineurin is maintained by two chaperones, cyclophilin A and FKBP12 [3]. Cyclosporine (CsA) and tacrolimus (FK506) inhibit calcineurin by binding cyclophilin A and FKBP12, respectively. These compounds target not only the mammalian but also the fungal homologues of calcineurin and therefore possess both, anti-rejection and antifungal activities. Targeting of calcineurin in *C. neoformans* with immunosuppressive inhibitors, or their non-immunosuppressive derivatives, combined with other antifungals, opens a new and promising avenue for drug development [4]. In fact, a synergistic effect of FK506 combined with bafilomycin A1, fluconazole, or caspofungin acetate has already been demonstrated *in vitro* [5].

In fungi, calcineurin confers resistance to environmental stresses, such as heat and high cation concentration. In *C. neoformans* functional calcineurin is required for growth at host physiological temperature and hyphal elongation during mating and monokaryotic fruiting. [6], [7]. Calcineurin affects cellular function by dephosphorylating its substrate proteins. In *C. neoformans*, the phospholipid binding protein Cts1 was identified as a calcineurin substrate during high temperature stress [8].

The major substrate of calcineurin in *S. cerevisiae* is the transcription factor Crz1 [9]. Following dephosphorylation, activated Crz1 translocates from the cytosol to the nucleus to trigger expression of genes with promoters that contain the calcineurin-dependent response element (CDRE) [9], [10]. *S. cerevisiae* Crz1 regulates genes involved in ion transport, cell wall synthesis and maintenance, lipid and sterol metabolism, vesicle transport and protein degradation [9]. Crz1 orthologs have been identified in human and plant fungal pathogens, where they are strongly associated with virulence and resistance to stress [11]–[16]. A homolog of Crz1 in *C. neoformans* designated Sp1 has recently been characterized [17]. Under conditions of glucose starvation, the gene expression profile of the *SP1* deletion mutant, *Δsp1,* closely correlated with the expression profile of a PKC deletion mutant, *Δpkc1*, but not with that of the calcineurin mutant, *Δcna1*. Moreover, overexpression of *SP1* partially restored cell wall integrity defects in *Δpkc1*. Based on these findings, [17] proposed that Crz1/Sp1 is regulated by Pkc1 phosphorylation in glucose-starved cells. However, potential regulation of Sp1 by calcineurin has not been tested under conditions other than glucose starvation.

Although calcineurin represents a promising target for anti-fungal drug development, surprisingly little is known about the mechanism of its activation in *C. neoformans,* or the identity of its downstream targets. Recently, Kozubowski *et al* reported that, in *C. neoformans,* GFP-tagged calcineurin accumulated in the bud neck of mother cells, and in puncta associated with the ER, following a rapid increase in environmental temperature [18], [19]. The GFP-tagged calcineurin co-localized with components of ER-Golgi trafficking machinery and with mRNA processing structures, identified as P-bodies and stress granules. In the present study we established *C. neoformans* Crz1/Sp1 (hereafter referred to as Crz1) as a *bona fide* ortholog of *S. cerevisiae* Crz1 by demonstrating its interaction with calcineurin and its calcineurin-dependent nuclear localization. Like other fungal CRZ proteins, *C. neoformans* Crz1 accumulated in nuclei in response to Ca^2+^ and physiological temperature. However, unlike its fungal counterparts, CnCrz1 migrated to cytosolic and nuclear puncta in response to heat shock and salt stress.

## Results

### Identification of the Crz1 Ortholog in *C. neoformans*

**Figure 1:**
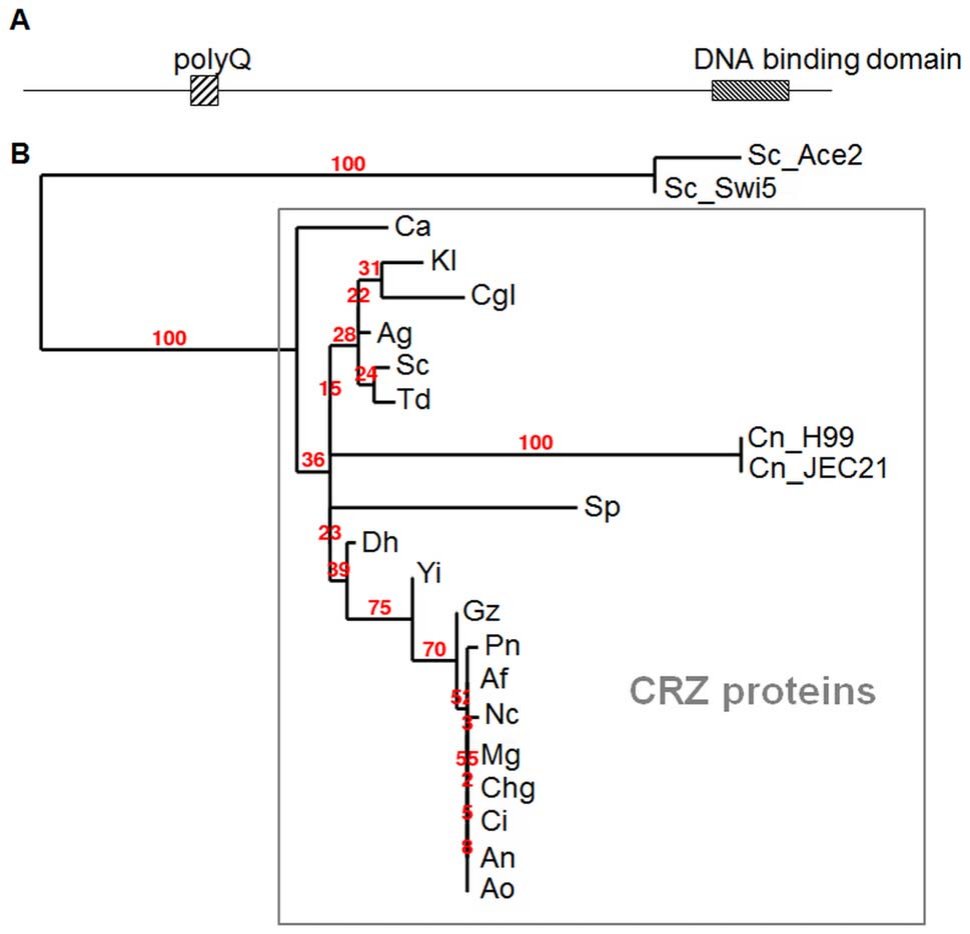
Sequence analysis of *C. neoformans* Crz1 and comparison with other fungal species. (A) Diagram of the Crz1 functional domains. (B) Phylogenetic tree of fungal CRZ proteins based on the alignment of their Zn finger DNA-binding domains. Bootstrap values are indicated. *S. cerevisiae* Swi5 and Ace2 represent transcription factors related to ScCrz1 Cn_H99, *Cryptococcus neoformans* serotype A; Cn_JEC21, XP_566613, *C. neoformans* serotype D; An, BAE94327, *Aspergillus nidulans*; Ao, BAE57003, *Aspergillus oryzae*; Af, EAL88401, *Aspergillus fumigatus*; Mo, XP_359644, *Magnaporthe oryzae*; Nc, EAA32849, *Neurospora crassa*; EAQ88414, Chg, *Chaetomium globosum*; Gz, XP_381517, *Gibberella zeae*; Ci, XP_001244584, *Coccidioides immitis*; Pn, EAT87393, *Phaeosphaeria nodorum*; Dh, CAG84727, *Debaryomyces hansenii*; Td, AAZ04388, *Torulaspora delbrueckii*; Ag, AAS51722, *Ashbya gossypii*; Sc, CAA95889, *Saccharomyces cerevisiae*; Kl, CAG99429, *Kluyveromyces lactis*; Yl, CAG80473, *Yarrowia lipolytica*; Ca, EAK97605, *Candida albicans*; Cgl, CAG62620, *Candida glabrata*; Sp, Q09838, *Schizosaccharomyces pombe*; Cd, CAX43071, *Candida dubliniensis*; Sc_Ace2, CAA97702, *Saccharomyces cerevisiae*; Sc_Swi5, CAA90369, *Saccharomyces cerevisiae*. Indicate what the red numbers mean.

The transcription factor, Crz1, has been identified as a calcineurin substrate in numerous filamentous fungi and yeast, including *S. cerevisiae* and *Candida albicans.* Fungal Crz1 orthologs share little sequence similarity, except at the C-terminus, which contains two or three zinc finger domains [15]. Using the zinc finger domains of *S. cerevisiae* Crz1 in a similarity search, we identified a putative Crz1 ortholog in the *C. neoformans* Serotype A genome. The predicted Crz1 protein (1094 amino acids, CNAG_00156), which contained three Zn finger domains, shares 94% identity with its homolog in *C. neoformans* serotype D, and, in the DNA binding domain, 44% identity with *S. cerevisiae* Crz1. While the C-terminal zinc finger-containing domain of *C. neoformans* Crz1 (*Cn*Crz1) is highly similar to other fungal CRZ proteins (Fig. 1A, S1), the N-terminal domain is unique to *C. neoformans*. We identified a short glutamine-rich region in *Cn*Crz1 which is also found in *S. cerevisiae* Crz1. Phylogenetic analysis performed using the conserved Zn finger DNA binding domain, positioned *Cn*Crz1 inside the cluster of Crz1 orthologs, while clearly separating them from their closest homologs represented by the *S. cerevisiae* transcription factors, Ace2 and Swi5 (Fig. 1B).

**Figure 2:**
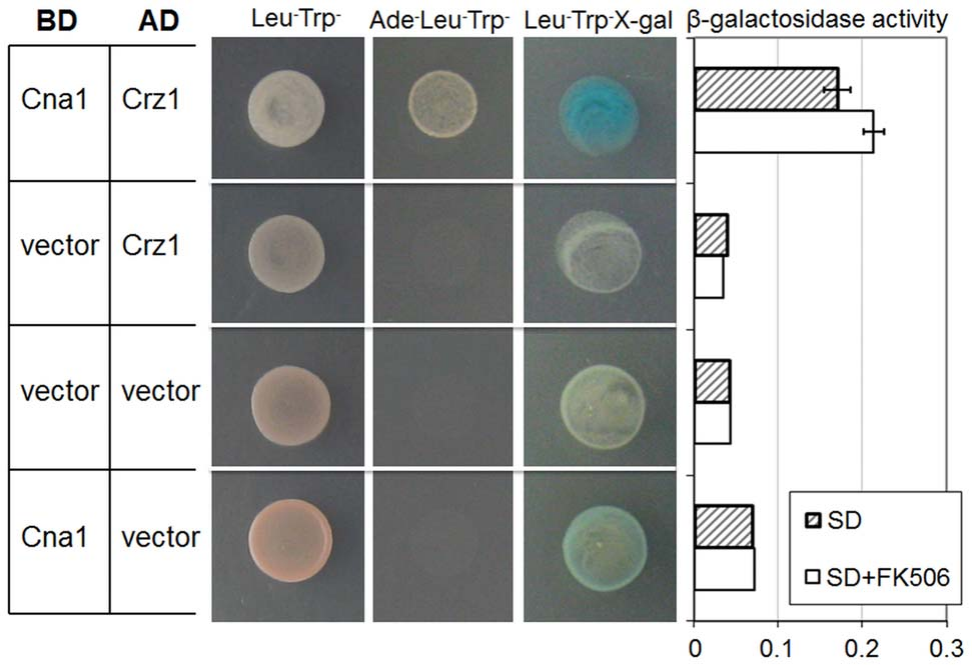
Yeast two hybrid analysis of Cna1 and Crz1 interaction. BD and AD designate GAL4 binding and activation domains fused to Cna1 and Crz1 respectively, where applicable. Vector equals empty vector. All combinations of transformants grew in the absence of Leu and Trp confirming plasmid retention in the parent strain. However, only yeast transformed with plasmids containing *CNA1* and *CRZ1* grew in the absence of Ade confirming an interaction which allows expression of Ade biosynthetic machinery. Quantitative analysis of β-galactosidase activity was performed using ONPG as a substrate. For this test, the cells were grown on SD Leu^-^Trp^-^ plates with or without 1 µg/ml FK506. Error bars represent standard deviation.

Yeast two hybrid analysis has been used to demonstrate the interaction of CRZ with subunit A of calcineurin in *S. cerevisiae* [20] and *Magnaporthe oryzae* [14]. We used the same strategy to investigate the interaction of *C. neoformans* Crz1 with calcineurin. Cna1 and Crz1 were fused to GAL4 binding (BD) and activation (AD) domains, respectively. Yeast expressing both *BD-CNA1* and *AD-CRZ1* grew in the absence of adenine and exhibited high β-galactosidase activity, demonstrating direct interaction between *C. neoformans* Cna1 and Crz1. Surprisingly, Cna1-Crz1 interaction was not disrupted in the presence of the calcineurin inhibitor, FK506, indicating that Cna1 interacts with Crz1 in its inactive state (Fig. 2).

### Cell Wall Integrity is Compromised in Crz1 and Cna1 Deletion Mutants

**Figure 3:**
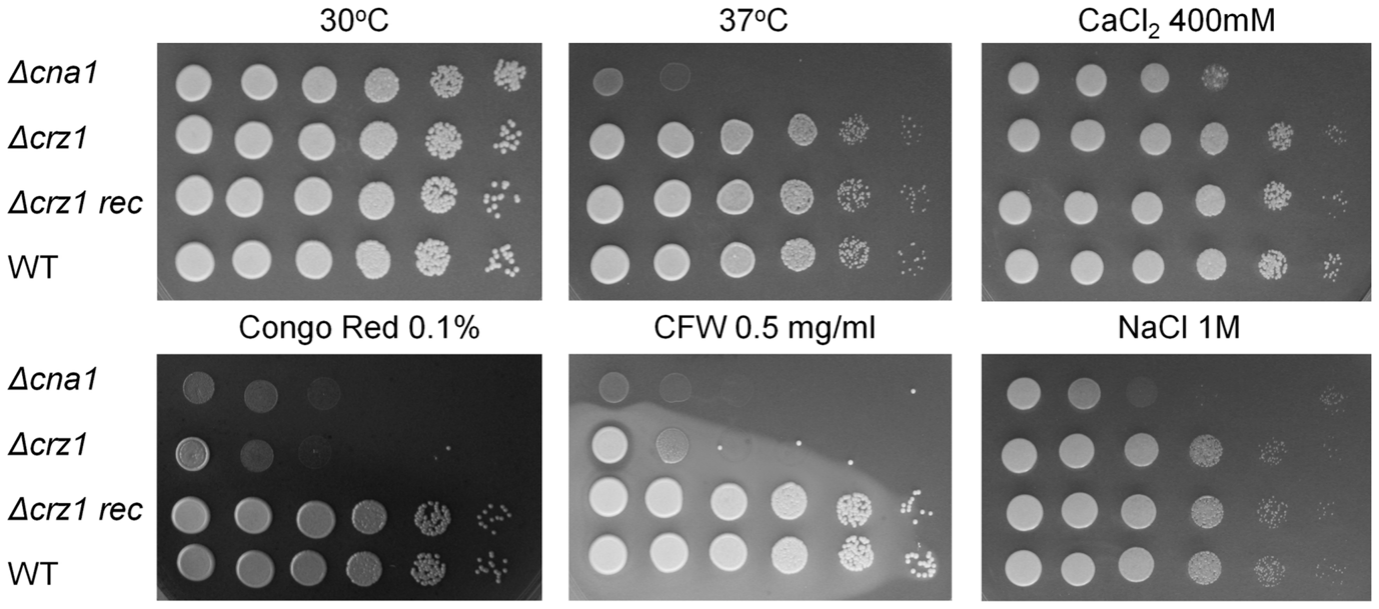
*Δcna1* and *Δcrz1* mutants share sensitivity to cell wall perturbing agents. WT H99, *Δcna1, Δcrz1 and Δcrz1-rec* (serially-diluted 10-fold, 10^6^–10 cells/spot from left to right) were spotted on YPD plates supplemented with NaCl, CaCl_2_, calcofluor white (CFW) or Congo Red as indicated, and incubated at 30°C. Thermosensitivity of the mutants was tested at 37°C on YPD plates.

To assess which of the *C. neoformans* calcineurin functions are relayed via Crz1, we deleted the *CRZ1* gene and compared the mutant phenotype with *Δcna1* (Fig. 3). The calcineurin-dependent phenotypes tested included growth at 37°C, cell wall integrity, susceptibility to ionic stress and production of mating filaments. Two other major virulence factors, capsule and melanin production, were also assessed. Growth of *Δcna1*, as compared to wild type, was compromised at human physiological temperature (37°C) and in the presence of CaCl_2_, NaCl, calcofluor white (CFW) and Congo red (Fig. 3). Of all the conditions tested, *Δcrz1* exhibited elevated sensitivity to the cell wall perturbing agents, CFW and Congo red. Reconstitution of the *Δcrz1* mutant with genomic *CRZ1* restored the *Δcrz1* phenotype back to that of WT (Fig. 3). Similar to the WT, *Δcrz1* produced capsule and melanin under inducing conditions, and, unlike *Δcna1*, formed abundant filaments during unilateral mating (Fig. S2).

### Nuclear Localization of CnCrz1 is Calcineurin-dependent

Fungal Crz1 shuttles between the cytosol and nucleus according to its activation state, and hence can be used as a reporter of calcineurin activity in response to environmental stimuli. In order to track the subcellular localization of Crz1, we replaced the endogenous *CRZ1* with a *CRZ1-GFP* gene fusion (Fig. S3). The resulting strain exhibited WT tolerance to CFW confirming that Crz1 function is not compromised by the addition of the C-terminal GFP tag (Fig. S4).

**Figure 4:**
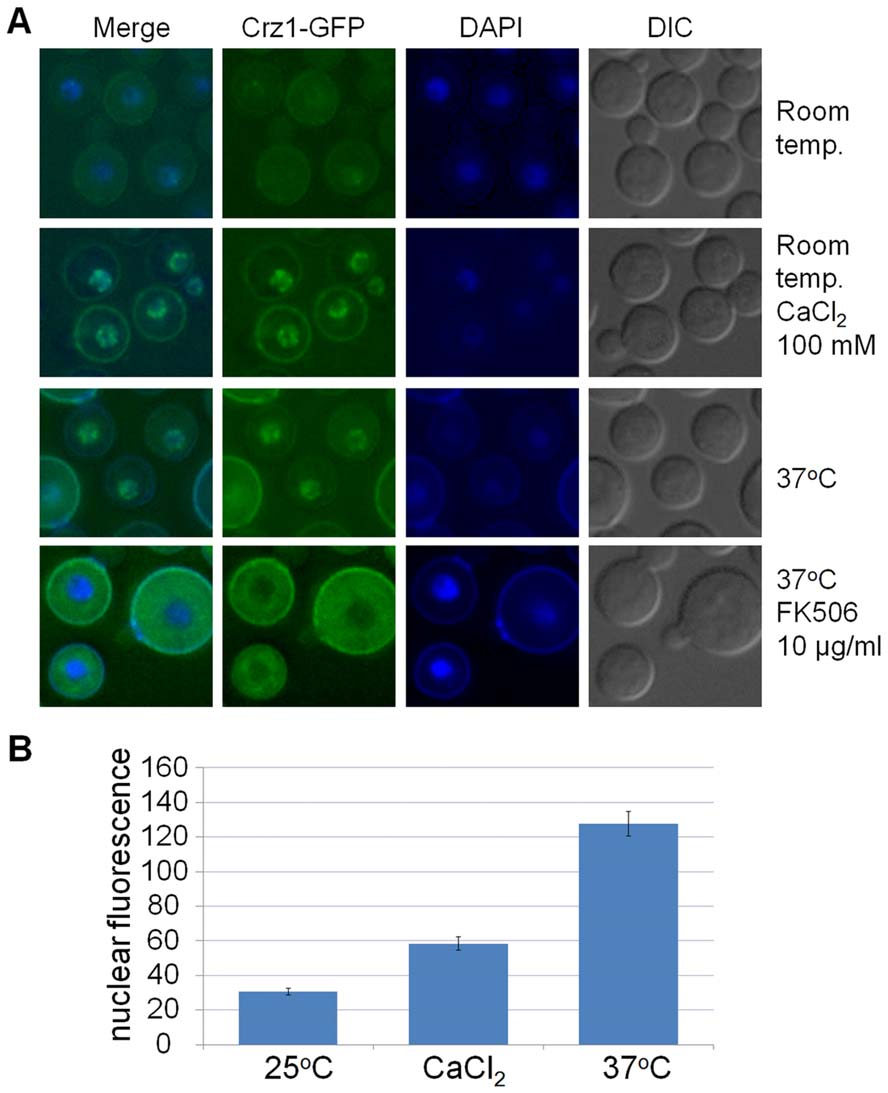
Nuclear targeting of Crz1-GFP is calcineurin-dependent and triggered by addition of calcium and elevated temperature. (A) Cells grown at room temperature show predominantly cytosolic Crz1-GFP localization. In cells treated with CaCl_2_ or grown at 37°C, Crz1-GFP is predominantly nuclear. The calcineurin inhibitor FK506 causes nuclear exclusion of Crz1-GFP at 37°C. (B) Quantification of the fluorescence intensity of nuclear Crz1-GFP. The differences between treated and untreated cells are statistically significant (n = 61 for 25°C control, n = 47 for CaCl_2_-treated cells and n = 50 for 37°C-grown culture; P<0.0001 using an unpaired two-tailed t-test with Welch correction). Error bars represent standard errors.

In cells grown to early exponential phase at room temperature (∼25°C), the Crz1-GFP fusion protein was distributed between cytosol and nuclei. However, in cells grown at higher temperatures (30–37°C), Crz1-GFP localized primarily in nuclei (Fig. 4). In some cells autofluorescence (visible as a ring around the cell) was comparable to the fluorescence of tagged Crz1 which is weak due to low *CRZ1* expression from the endogenous promoter. To confirm the dependency of CnCrz1 subcellular localization on calcineurin activity, we added the calcineurin inhibitor, FK506, to the culture grown at 37°C. Within 1 hour of FK506 treatment, Crz1-GFP was completely excluded from nuclei (Fig. 4). In filamentous fungi and *S. cerevisia*e, Crz1 also translocates to nuclei in response to extracellular calcium [10], [14], [21], [22]. Similarly, we found that addition of 100–300 mM CaCl_2_ to *C. neoformans* cells grown at 25°C triggered nuclear localization of Crz1 (Fig. 4). The effect of 37°C and calcium addition on the intensity of Crz1-GFP fluorescence associated with nuclei was also quantified (Fig. 4B), and the results confirm the observations in Figure 4A. These findings are consistent with CnCrz1 functioning downstream of calcineurin and establish CnCrz1 as an ortholog of Crz1 of *S. cerevisiae* and other fungi.

### Calcineurin/Crz1 Induces Expression of Chitin Synthase-encoding CHS6 in Response to Cell Wall Stress

**Figure 5:**
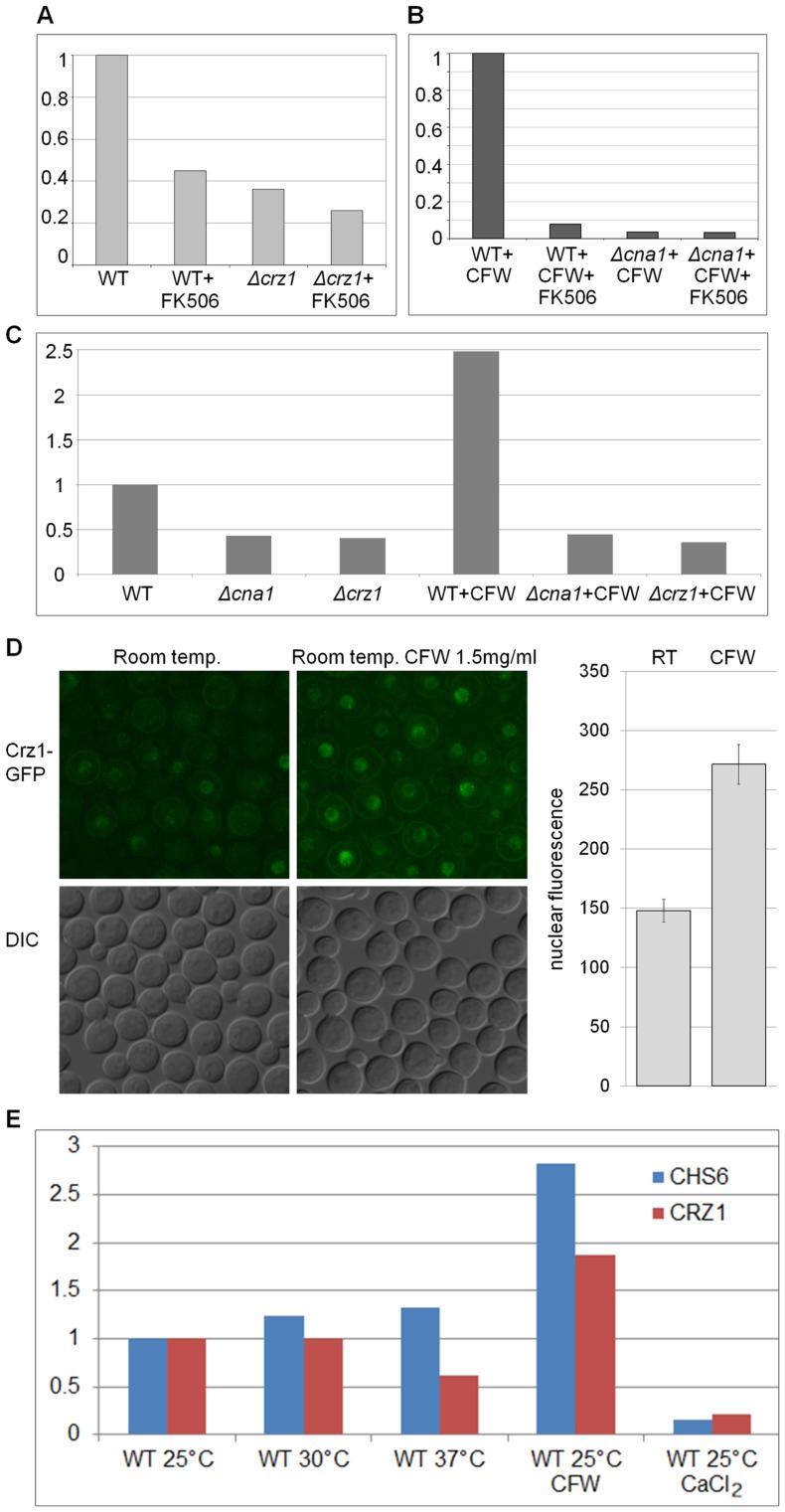
Calcofluor white triggers calcineurin/Crz1-dependent expression of chitin synthase *CHS6* and nuclear accumulation of Crz1-GFP. (A) Expression of *CHS6* in YPD-grown WT and *Δcrz1* with or without FK506 (10 µg/ml). The bar graph represents sqPCR data and is the average of two independent experiments. (B) Expression of *CHS6* in WT and *Δcna1* grown in YPD and exposed to CFW (2.2 mg/ml) or CFW+FK506 (10 µg/ml) for 1 hour. *CHS6* expression was quantified by qPCR (3 technical replicates). (C) Induction of *CHS6* expression in YPD grown cultures following exposure to CFW (1 hour treatment, 1.5 mg/ml). The bar graph represents sqPCR data and is the average of 4 independent experiments. The actin gene (*ACT1*) was used for normalization in all the expression studies. Y-axis values represent expression relative to untreated WT. (D) Nuclear accumulation of Crz1-GFP following CFW treatment (1 hour, 1.5 mg/ml) is shown on a micrograph. The bar graph represents quantification of nuclear fluorescence. The difference between treated and untreated cells is statistically significant (n = 50, P<0.0001 in an unpaired two-tailed t-test with Welch correction). The error bars represent standard error. (E) Expression of *CRZ1* and *CHS6* was tested under different temperature conditions and in the presence of 200 mM CaCl_2_ or 2.5 mg/ml CFW (1 hour exposure) by qPCR (3 technical repeats).

Several lines of evidence prompted us to test whether the chitin synthase gene, *CHS6* (CNAG_00546), is a potential target of calcineurin signaling: first, both *Δcna1* and *Δcrz1* are sensitive to CFW, a cell wall perturbing agent that binds chitin and blocks formation of the fibrillar lattice [23]; second, *CHS6* is induced in *C. neoformans* grown at 37°C, conditions under which calcineurin is essential for viability [24], and third, chitin synthase genes are regulated by calcineurin and Crz1 in *S. cerevisiae* and *Magnaporthe oryzae* [9], [14]. Treatment of *C. neoformans* with FK506 caused a marked reduction in *CHS6* expression in WT, but not in *Δcna1* or *Δcrz1* (Fig. 5A and B), consistent with *CHS6* being regulated by calcineurin via Crz1. The reduction of *CHS6* expression in WT following addition of FK506 was even more pronounced in the presence of CFW. We therefore tested expression of *CHS6* in WT, *Δcna1* and *Δcrz1* following addition of CFW. In untreated cultures, expression of *CHS6* was consistently lower in both mutants, as compared to wild type (Fig. 5C). CFW treatment triggered, on average, a 2.5-fold induction of *CHS6* in wild type, but not in the mutants, where the *CHS6* expression levels remained similar to those of untreated cells. These results suggest that *CHS6* is regulated by the calcineurin/Crz1 signaling pathway under normal and cell wall stress conditions. Furthermore, in agreement with this finding, CFW caused a moderate increase in the amount of nuclear Crz1-GFP (Fig. 5D).

We also tested the expression of *CHS6* and *CRZ1* under different temperatures, during calcium-induced and cell wall stress. Surprisingly, expression of *CRZ1* was down-regulated at 37°C and in the presence of calcium (Fig. 5E), conditions which trigger nuclear targeting of Crz1 (Fig. 4). However, following exposure to CFW, *CRZ1* expression was up-regulated, similar to *CHS6*. In the presence of calcium, expression of *CHS6* was markedly reduced, indicating that nuclear localization of Crz1 is not sufficient to induce *CHS6* expression.

### Differential Response of Crz1 to Stress

**Figure 6:**
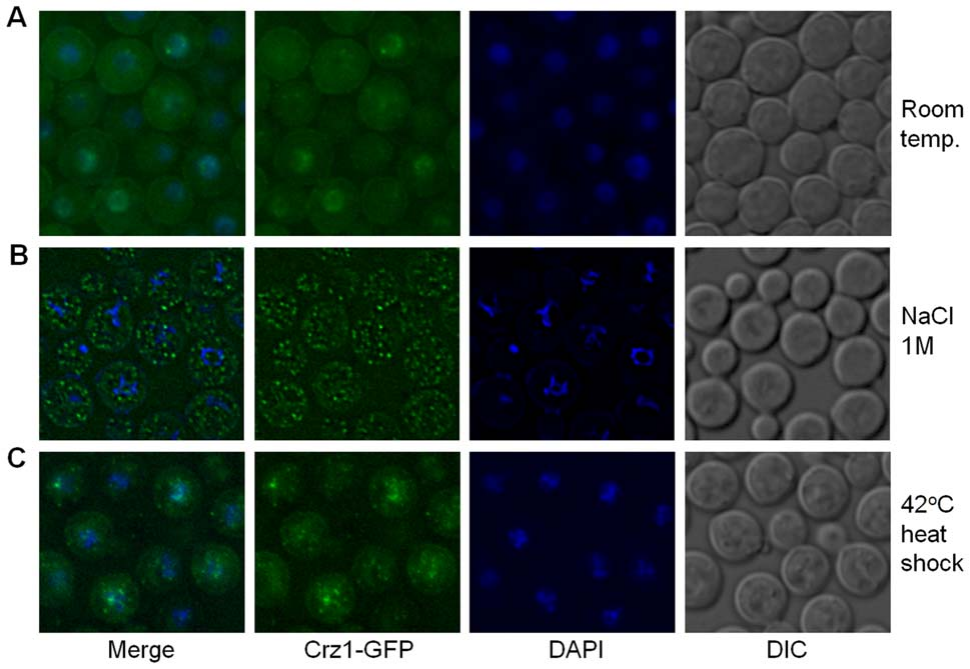
Salt stress and high temperature trigger punctate localization of Crz1-GFP. (A) Cells grown at room temperature show predominantly cytosolic and occasional nuclear Crz1-GFP localization, while Crz1-GFP is sequestered in cytosolic puncta in cells treated with 1 M NaCl (B). In cells exposed to 42°C, puncta are localized in and around nuclei (C).

**Figure 7:**
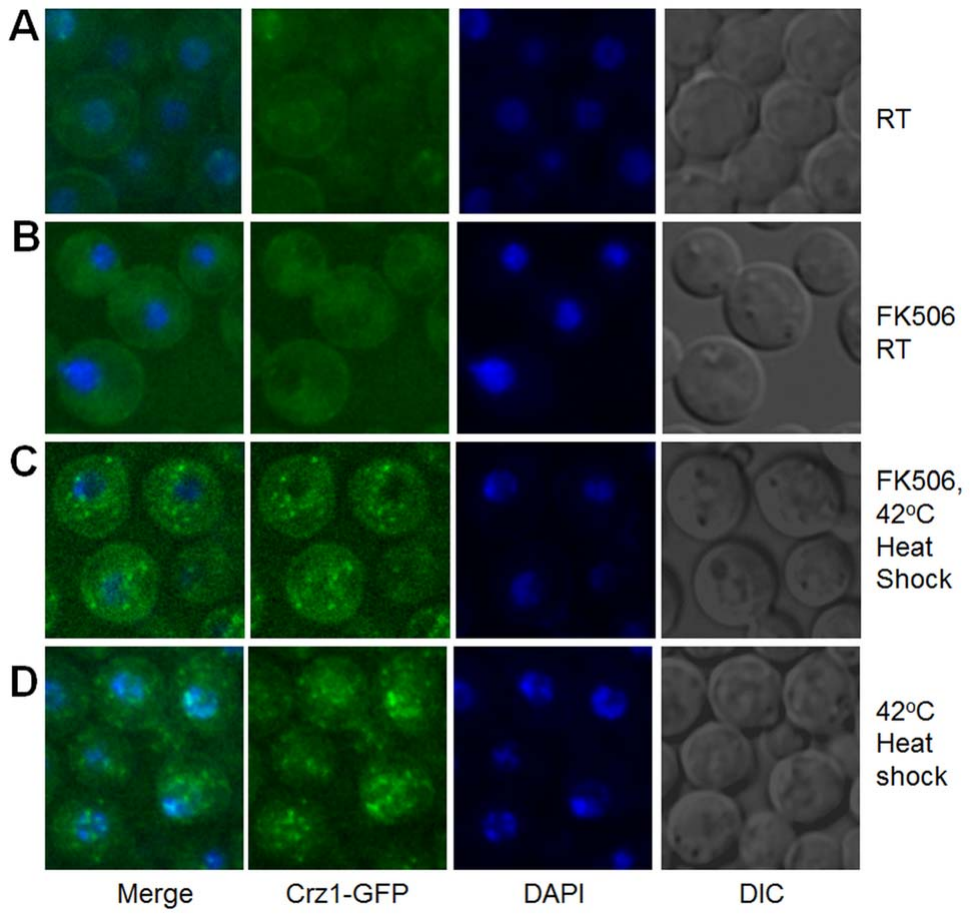
FK506 causes exclusion of heat shock-induced Crz1-GFP puncta from the nucleus. *C. neoformans* cells growing at room temperature (A) were treated with 10 µg/ml FK506 for 1 hour (B) and then exposed to 42°C for 5 min. The fluorescence pattern was compared in FK506-treated and untreated cells within 45 minutes following heat shock (C, D).

We investigated the subcellular localization of Crz1-GFP in response to severe stress caused by exposure of *C. neoformans* to 1 M NaCl or to heat shock at 42°C for 5 minutes. Unexpectedly, these treatments caused Crz1-GFP to localize to distinct cytosolic and nuclear puncta (Fig. 6 and Fig. S5). This was in contrast to the translocation of Crz1-GFP to the nucleus which we observed with continuous incubation at 37°C, or the addition of extracellular calcium (Fig. 4 and Fig. S5). Salt-induced puncta were distributed evenly in the cytosol, while heat stress-induced puncta were more centered in and around the nucleus. The transient nature of these fluorescent puncta was indicated by their gradual disappearance following the removal of salt where, by one hour post-washing, all puncta had disappeared. These puncta were not observed if the cells were gradually acclimatized to increasing concentrations of NaCl. Importantly, addition of FK506 did not interfere with formation of puncta in response to NaCl (not shown) and heat shock, but caused nuclear exclusion of Crz1-GFP in stressed, as well as in non-stressed cells (Fig. 7). These findings indicate that association of Czr1-GFP with puncta occurs independently of calcineurin activity.

### Calcineurin Protein is not Required for Sequestration of Crz1 to Cytosolic Puncta

**Figure 8:**
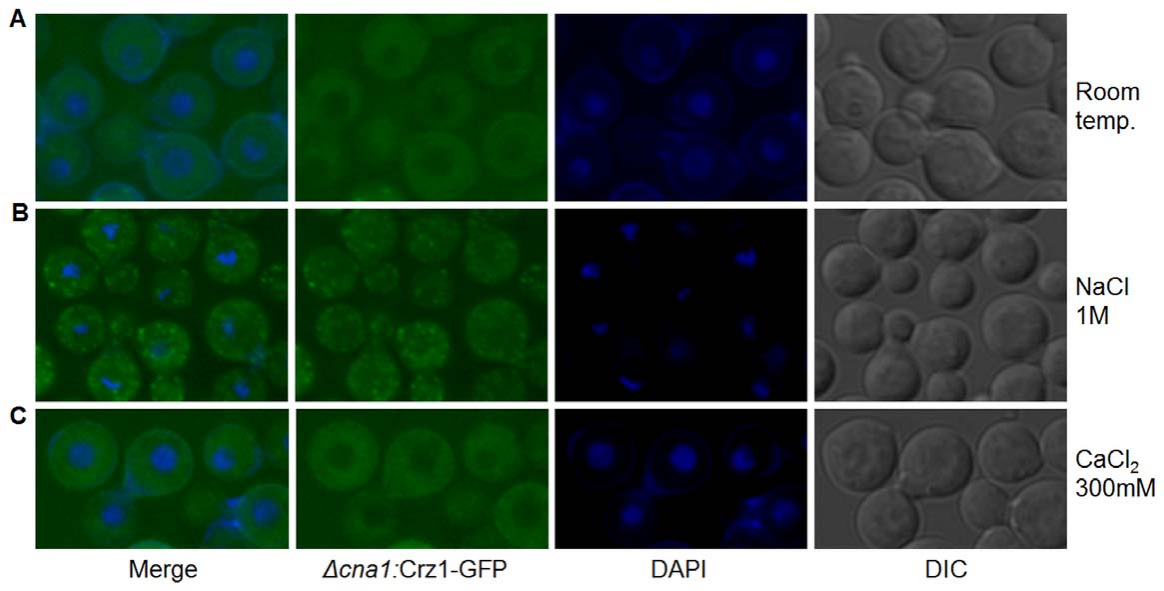
Calcineurin protein is required for targeting of Crz1-GFP to the nuclei, but not to the cytosolic puncta. The strain used in all panels is Crz1-GFP:*Δcna1.* (A) Crz1-GFP is excluded from the nuclei in *Δcna1* grown at room temperature; (B) 1 M NaCl triggers punctate Crz1-GFP localization even in the absence of calcineurin; (C) addition of calcium fails to trigger nuclear translocation of Crz1-GFP in *Δcna1*.

Calcineurin is known to have functions that are dependent and independent of its phosphatase activity [25]. We therefore considered the possibility that, independent of its catalytic activity, calcineurin protein is required for Crz1 sequestration to cytosolic puncta upon exposure to salt or heat stress. We therefore tracked the *Crz1-GPF* reporter in the calcineurin deletion mutant. As expected, Crz1-GFP was excluded from the nuclei under normal growth conditions (room temperature), and following addition of 300 mM CaCl_2_ or CFW (Fig. 8 and Fig. S6, respectively). However, upon exposure of the cells to 1 M NaCl, Crz1-GFP formed puncta closely resembling those formed in the WT strain with a functional Cna1 (Fig. 8). These findings suggest that Crz1-GFP responds differentially to two different types of stimuli. A stimulus of the first type, which proceeds via Ca^2+^/calcineurin, triggers Crz1-GFP nuclear translocation. A stimulus of the second type, which proceeds independently of calcineurin, causes Crz1-GFP to accumulate in punctate structures. Heat shock is likely to provide a combination of two stimuli causing Crz1-GFP to form puncta in and around the nucleus.

### Crz1-GFP does not Co-localize with Stress Granules Formed Under Heat and Salt Stress

**Figure 9:**
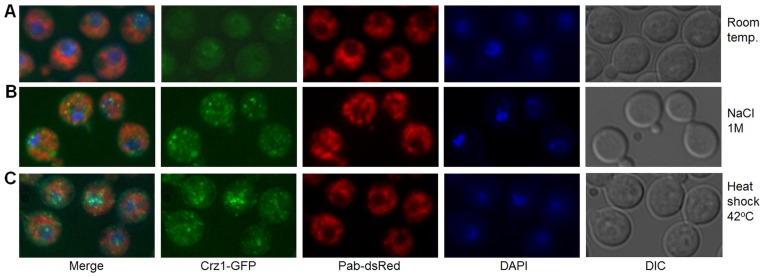
Crz1-GFP does not co-localize with Pab-dsRed, a marker of stress granules. (A) Under non-stressful conditions, Pab-dsRed fluorescence concentrates around the nuclei and in the cytosol; (B) salt stress triggers accumulation of Pab-dsRed in cytosolic puncta; (C) Pab-dsRed distribution is partially disrupted following heat shock.

In *C. neoformans,* calcineurin has been reported to respond to a rapid temperature shift (from 25°C to 37°C) by co-localizing with ER-Golgi trafficking machinery and mRNA processing structures in puncta centered around the nucleus. These mRNA processing structures were identified as P-bodies and stress granules [18]. P-bodies are considered to be focal points for mRNA degradation. They are present in unstressed cells, but increase in number following stress. In contrast, stress granules are formed specifically in response to severe stress, similar to the Crz1-GFP puncta shown in Figure 6. This prompted us to investigate stress granules as a potential destination of Crz1-GFP following severe stress. In order to visualize stress granules, we tagged the polyA-binding protein marker (Pab) with dsRed. In the absence of stress, Pab-dsRed fluorescence was visible as a tight ring around the nucleus and was evenly distributed throughout the cytosol. Following NaCl treatment or heat shock, Pab-dsRed distribution became patchy with a few peaks of more intense fluorescence (Fig. 9). However, these peaks only occasionally overlapped with Crz1-GFP puncta, ruling out targeting of Crz1 to stress granules (Fig. S7).

### Potential Calcineurin Docking Domain in *Cn*Crz1

Similar to other calcineurin substrates, *S. cerevisiae* Crz1 contains a calcineurin docking domain (CDD), or PxIxIT motif, which was first characterized in the mammalian NFAT family of calcineurin-regulated transcription factors [26]. We attempted to identify the PxIxIT motif in *Cn*Crz1 based on the location and sequence similarity in relation to the confirmed PIISIQ motif in *S. cerevisiae* and the predicted CDD sequences of other fungal CRZs. However, the best candidate, ^659^PRLDPD^664^, is not predicted to constitute a β-sheet which is crucial for interaction with calcineurin [27], [28]. We therefore mutagenized two other candidate motifs, ^451^PMICIQ^456^ and ^394^PNIVTQ^399^ to PMIddQ and PNddTQ respectively, eliminating their predicted β-sheet configuration. However, these modifications did not affect nuclear targeting of the mutant proteins and the formation of fluorescent puncta (our unpublished data).

## Discussion

In this study we have demonstrated that *Cn*Crz1 is activated by calcineurin in response to physiological temperature, extracellular calcium and cell wall perturbation, as manifested by its nuclear localization under these conditions. Recently, [17] demonstrated that Crz1 is regulated by PKC under starvation stress and consequently, they referred to Crz1 as Sp1 based on its homology to the Pkc1-dependent specificity protein-1 (Sp1) transcription factors of metazoans. Similar to other fungal CRZ proteins and to the findings of [17], we identified a conserved C-terminal DNA binding domain in *Cn*Crz1 containing three zinc finger motifs. Although alignment of each of the motifs with homologous motifs of SP1 transcription factors showed significant similarity [17], phylogenetic analysis performed using the entire DNA binding domain containing all three Zn-finger motifs demonstrated that *Cn*Crz1 is an ortholog of fungal Crz1 proteins. The results of the two studies support a model whereby *Cn*Crz1/Sp1 is under dual regulation: under non starvation stress it responds to calcineurin, and under starvation stress it is affected by Pkc1, to restore cell wall integrity.

Deletion of the *CRZ1* gene rendered *C. neoformans* cells sensitive to cell wall perturbing agents, but did not compromise their ability to grow at host physiological temperature, form mating filaments, or tolerate cations. Taken together these findings suggest that Crz1 is responsible for relaying calcineurin functions related to cell wall integrity. It appears that the role of Crz1 in thermotomerance has been superceded at some point in the evolutionary process by other transcription factors that still remain to be identified. In the pathogenic yeasts, *Candida albicans* and *C. dubliniensis,* Crz1 performs only some functions of calcineurin. For example, growth at high Ca^2+^ concentrations, but not resistance to CFW and caspofungin, is regulated by calcineurin via Crz1 [29], while in *S. pombe, Δcrz1* phenotype differs dramatically from the phenotype of the calcineurin deletion mutant [22]. In contrast, Crz1 and calcineurin mutant phenotypes are very similar in *S. cerevisiae* [30], suggesting that Crz1 is more central to the function of calcineurin in this non-pathogenic yeast.

Despite the species-specific differences in Crz1 function and the low sequence homology among Crz1 proteins, several gene targets are conserved across species. For example in *S. cerevisiae,* calcineurin/Crz1 regulates expression of genes encoding ion pumps required for distinct aspects of ion homeostasis: *PMC1* encodes a Ca^2+^-ATPase responsible for calcium sequestration in vacuoles, and *PMR1* similarly transports Ca^2+^ and Mn^2+^ into the Golgi; *ENA1*, *ENA2* and *ENA5* encode plasma membrane Na^+^-ATPase exporters [9]. Orthologs of these genes are also regulated by calcineurin and Crz1 in *C. albicans* [21], *C. dubliniensis* [29], *M. oryzae* [14] and *A. fumigatus* [13]. Genes encoding chitin synthases (*CHS*), which are essential for maintaining cell wall integrity, are regulated by calcineurin and Crz1 in *C. albicans* and *S. cerevisiae*, and in the filamentous fungi *M. oryzae* and *A. nidulans* [9], [14], [15], [31], [32]. Our study shows that, similar to other fungal species, expression of *CHS6* in *C. neoformans* requires calcineurin and Crz1 under both normal conditions and CFW-induced stress.

In *C. neoformans* cells grown at room temperature, Crz1-GFP was distributed diffusely throughout the cytosol, while being only slightly enriched in some of the nuclei. In both *Δcna1* and the WT incubated with the calcineurin inhibitor FK506, Crz1 was completely excluded from nuclei even under non-inducing conditions, suggesting that calcineurin maintains a basal level of Crz1 activation. Surprisingly, nuclear accumulation of Crz1-GFP was induced by increasing the growth temperature from 25°C to 30°C, the condition under which we observed the highest growth rate (our unpublished observation). This apparent paradox may be explained by the need to increase Crz1 activity in order to sustain cell wall biogenesis during rapid growth.

Crz1-GFP was activated and localized mainly in nuclei during growth at 37°C. However, despite Crz1 activation, it was dispensable for growth at this temperature (Fig. 3). Similarly, Crz1 was dispensable for growth in the presence of 400 mM CaCl_2_, despite having translocated to the nucleus. The phenomenon of activation without apparent function has been described for *S. pombe* Prz1 where activity of the reporter gene fused to the calcineurin/Prz1-dependent response element, was increased 19-fold following exposure to 0.3 M KCl [33]. However, unlike the calcineurin-deficient mutant, *Δprz1* was resistant to the ionic stress caused by KCl [22]. Positive correlation between *C. neoformans* Crz1 nuclear accumulation and the ensuing physiological response was observed in the case of cell wall perturbation by CFW, where a modest increase in Crz1 nuclear localization correlated with the induction of the Crz1-dependent chitin synthase gene *CHS6*, followed by establishment of Crz1-mediated tolerance to CFW and Congo red. In accordance with its importance in maintaining cell wall integrity, *CnCRZ1* expression was up-regulated in the presence of calcofluor white. However, *CnCRZ1* was down-regulated in response to calcium, in contrast to *C. albicans CRZ1* [21]. This difference correlates with the importance of Crz1 for tolerance to calcium-induced stress in *C. albicans*, but not in *C. neoformans*.

In contrast to nuclear translocation of Crz1 in cultures growing at 37°C, heat shock (5 min at 42°C) and exposure to high concentrations of NaCl resulted in Crz1 localization in cytosolic and nuclear puncta. Targeting of Crz1-GFP into cytosolic puncta did not require calcineurin since addition of FK506 or deletion of *CNA1* failed to prevent their formation. However, addition of FK506 caused exclusion of puncta from nuclei, suggesting that the nuclear localization of Crz1 following heat shock is calcineurin-dependent. Taken together, these findings suggest that *C. neoformans* Crz1 accumulates in nuclei in response to a Ca^2+^/calcineurin-mediated signal, whereas its localization in cytosolic puncta is calcineurin-independent.

Interestingly, it was recently shown that *C. neoformans* calcineurin is targeted to ER-associated puncta in response to a rapid temperature shift from 24°C to 37°C, and that this targeting does not require its phosphatase activity [8], [18], [19]. Moreover, *C. neoformans* calcineurin accumulated in cytosolic puncta in response to high salt [18]. Some of the puncta formed in response to the temperature shift co-localized with mRNA processing structures (stress granules and P-bodies) and with components of the ER-Golgi trafficking machinery [18]. We did not observe significant overlap between fluorescence of Crz1-GFP and the stress granule marker Pab following heat shock or high salt exposure, consistent with calcineurin and Crz1 being in separate compartments under these stress conditions.

Although a rapid temperature shift from 24°C to 37°C caused punctate calcineurin localization, in cells that had adjusted to the elevated temperature (after 45 minutes incubation at 37°C), calcineurin assumed a diffuse cytosolic distribution [18]. Since Crz1 is nuclear in cells that have been continuously grown at 37°C, it is likely to be activated and directed to the nucleus by the diffusely-distributed calcineurin. Puncta-associated calcineurin may therefore affect different calcineurin substrates. In support of this hypothesis, phospholipid-binding protein, Cts1 (another calcineurin substrate), co-localized with calcineurin in cytosolic puncta and also co-immunoprecipitated with Cna1, with greater complex formation at 37°C compared to 24°C, suggesting that Cts1 is a substrate of calcineurin during high temperature stress [8].

Calcineurin-independent CRZ signaling has been reported in fungi. For example, in *C. dubliniensis*, Crz1 regulates a thigmotropic (surface sensing) response independently of calcineurin [29]. Similarly, in *C. neoformans*, Crz1 is involved in response to glucose starvation independently of calcineurin [17]. It is possible that in glucose-starved cells calcineurin, at least in part, migrates to stress granules and P-bodies, which are known to form in yeast cells under these conditions [34], [35] thereby relinquishing control over Crz1. Consistent with this hypothesis, Crz1 regulates a set of calcineurin-independent genes in conjunction with Pkc1 under conditions of glucose starvation [17].

Crz1-GFP puncta formation may be a part of a stress adaptation mechanism, since this phenomenon is only temporary and the puncta disappear following adaptation to a new condition. Alternatively, it could be a manifestation of the partial unfolding of Crz1 during severe stress and the subsequent aggregation of unfolded protein into large protein complexes. In support of this hypothesis, Crz1 is dispensable for high salt tolerance, a condition where we demonstrated the formation of multiple Crz1-associated cytosolic puncta. Moreover, Crz1 contains a short polyglutamine motif and polyglutamines are known to promote protein aggregation, especially in response to stress [36].

In summary we have shown that Crz1 is a *bona fide* target of calcineurin in *C. neoformans* and have identified unique features of this transcription factor. To our knowledge Crz1 of *C. neoformans* is the only fungal homologue reported to manifest stimulus-specific calcineurin-dependent and independent responses to environmental stresses. The physiological significance of the punctate Crz1 distribution and the identity of the target structures remain to be determined.

## Materials and Methods

### Strains and Media

Wild-type *C. neoformans* var. *grubii* strain H99 (serotype A, *MAT*a) was used in this study (WT). The strains Crz1-GFP, *Δcrz1*, Crz1-GFP:Pab-dsRed were created from H99 (Fig. S3). The calcineurin (*Δcna1*) and G-protein α subunit (*Δgpa1*) deletion strains were kindly provided by Joseph Heitman and Andy Alspaugh, respectively (Duke University, Durham, NC, USA). The KUTAP vector containing GFP optimized for fluorescence in *C. neoformans* was a gift from Peter Williamson (NIAID, NIH, Bethesda,MA, USA). All strains were routinely cultured on YPD medium (1% yeast extract, 2% peptone and 2% dextrose). L-DOPA agar (1 g/l L-asparagine, 0.5 g/l MgSO_4_, 3 g/l KH_2_PO_4_, 1 mg/l thiamine, 1 mM L-DOPA) was used to test melanization. Capsules were induced on RPMI agar (RPMI, 2% glucose, 0.165 M MOPS pH 7, 1.5% bacto agar, 0.03% glutamine) by incubation at 37°C in a 5% CO_2_ atmosphere for 3 days. V8 mating medium was prepared as described previously (23).

### *C. neoformans* CRZ1 Identification and Comparison with Other Species

To identify a putative *C. neoformans* Crz1 ortholog, the H99 genome (http://www.broadinstitute.org/annotation/genome/cryptococcus_neoformans/Blast.html) was searched with the C-terminal sequence of *S. cerevisiae* Crz1. CNAG_00156 showed significant homology to the *S. cerevisiae* gene and was designated *CnCRZ1*. The following fungal proteins were used for the alignment: Cn_H99, Cryptococcus neoformans serotype A; Cn_JEC21, XP_566613, *C. neoformans* serotype D; An, BAE94327, *Aspergillus nidulans*; Ao, BAE57003, *Aspergillus oryzae*; Af, EAL88401, *Aspergillus fumigatus*; Mo, XP_359644, *Magnaporthe oryzae*; Nc, EAA32849, *Neurospora crassa*; EAQ88414, Chg, *Chaetomium globosum*; Gz, XP_381517, *Gibberella zeae*; Ci, XP_001244584, *Coccidioides immitis*; Pn, EAT87393, *Phaeosphaeria nodorum*; Dh, CAG84727, *Debaryomyces hansenii*; Td, AAZ04388, *Torulaspora delbrueckii*; Ag, AAS51722, *Ashbya gossypii*; Sc, CAA95889, *Saccharomyces cerevisiae*; Kl, CAG99429, *Kluyveromyces lactis*; Yl, CAG80473, *Yarrowia lipolytica*; Ca, EAK97605, *Candida albicans*; Cgl, CAG62620, *Candida glabrata*; Sp, Q09838, *Schizosaccharomyces pombe*; Cd, CAX43071, *Candida dubliniensis*; Sc_Ace2, CAA97702, *Saccharomyces cerevisiae*; Sc_Swi5, CAA90369, *Saccharomyces cerevisiae*. Zinc finger domain sequences were manually extracted from the alignment and used for phylogenetic analysis (Fig. S1). The analysis was performed on the Phylogeny.fr platform. The phylogenetic tree was reconstructed using the maximum likelihood method [37].

### Yeast Two Hybrid Analysis

*CRZ1* and *CNA1* were PCR amplified from *C. neoformans* cDNA using primers listed in Table S1 and cloned into pGADT7 (prey) and pGBKT7 (bait) vectors (Clontech), respectively. Growth of AH109 yeast (Clontech) expressing both bait and prey vectors was tested on SD medium lacking leucine and tryptophan to select for each vector, and histidine and adenine to test for an interaction. Empty pGBKT7 and pGADT7 were used as negative controls. Since Cna1 fused to the GAL4 binding domain exhibited background activation of the *HIS3* reporter, only *ADE2* and *lacZ* reporter genes were used to test Crz1-Cna1 interaction. LacZ activity was assayed using the X-gal overlay method [38]. To quantify β-galactosidase activity, yeast cells were grown overnight on SD plates without leucine and tryptophan supplemented with 1 µg/ml FK506. The ONPG liquid culture assay was performed as described in the Clontech Yeast Protocol Handbook, using cells collected from plates rather than YPD broth cultures.

### Generation of Transgenic Strains Via Double Cross-over Recombination

Plasmids pJAF (containing a Neomycin resistance cassette, NEO^R^) and pCH233 (containing a Nourseothricin resistance cassette, NAT^R^) were kindly provided by Dr John R Perfect, Duke University, Durham, NC, USA. A *CRZ1* gene deletion construct was made by overlap PCR, joining 5′ flank (1092 bp sequence upstream of *CRZ1*), NEO^R^ from pJAF (*ACT1* promoter, neomycin phosphotransferase, *TRP1* terminator) and 3′ flank (1049 bp downstream of *CRZ1*) (see Table S1 for primer sequences and Fig. S3 for the recombination diagram). A CRZ1-GFP construct designed to introduce *GFP* at the 3′ end of the endogenous *CRZ1* gene was generated by joining the 5′ flank (last 1148 bp of the *CRZ1* gene excluding the stop codon), *GFP* and the elongation factor 1 terminator (*EF1T*) from KUTAP, NEO^R^, and the 3′ flank (950 bp downstream of *CRZ1*) (Table S1, Fig. S3). For *Δcrz1* reconstitution, the antibiotic resistance of *Δcrz1* was changed from NEO to NAT by transforming *Δcrz1*(NEO^R^) with a NAT resistance cassette, creating *Δcrz1*(NAT^R^). The genomic *CRZ1* sequence including the promoter and ORF (4969 bp) was fused to the GFP-EF1T-NEO^R^ fragment in the pCR2.1 cloning vector (Life Technologies). Linearized plasmid was transformed into *Δcrz1*(NAT^R^) to allow ectopic integration of the CRZ1-GFP-EF1T-NEO^R^ fragment into the genome. A PAB-dsRED vector designed to introduce *dsRED-Express* (Clontech) at the 3′ end of the endogenous *PAB* gene (CNAG_04441) was generated by joining the 5′ flank (last 945 bp of *PAB* gene excluding stop codon), *dsRED*, *EF1* terminator (from KUTAP), NAT^R^ from pCH233 (*ACT1* promoter, nourseothricin acetyltransferase, *TRP1* terminator), and the 3′ flank (1108 bp downstream of *PAB*).

Transformation of each of the constructs described above was carried out using the biolistic method [39]. NEO or NAT resistant transformants were selected on YPD agar plates supplemented with 0.5 M sorbitol and 200 µg/ml G418 or 100 µg/ml Nourseothricin, respectively. Correctly targeted integration of each of the constructs was confirmed by PCR amplification across the junction point of integration of the vector with genomic DNA, using a forward primer that anneals outside the region of integration and a reverse primer that anneals within the construct. (Fig. S3). Potential contamination of positive transformants with WT was ruled out by performing a PCR with primers annealing specifically to the region of the gene being deleted and demonstrating absence of a PCR product of the expected size and by the absence of gene expression using RT-PCR. To test the expression of recombinant genes, we isolated RNA from the YPD-grown cultures: cell pellets were disrupted with glass beads in the presence of Trizol reagent (Ambion) using a beat-beater. RNA was subsequently extracted according to manufacturer’s instructions. RNA was treated with DNAse I and cDNA was synthesized using Invitrogen SuperScript II cDNA synthesis kit.

### Gene Expression

To test chitin synthase *CHS6* expression (Fig. 7A, B, C) the cells were cultured in YPD overnight at 30°C and diluted to OD 0.5 in the morning. After an additional 4 hours incubation, the cells were treated with CFW (1.5–2.2 mg/ml, as indicated) and/or FK506 (10 µg/ml) for 1 hour. To test the expression of *CRZ1* and *CHS6* under different stress conditions (Fig. 7E), the cells were grown overnight at 25°C, diluted to OD = 0.5 and incubated for 5 hours at 25°C, 30°C and 37°C. For the cell wall and ionic stresses, cells which had been grown at 25°C were supplemented with 2.5 mg/ml CFW or 200 mM CaCl_2_ for 1 hour. The cells were collected, snap-frozen and RNA extraction and cDNA synthesis were performed as described above. Semi-quantitative and real time PCR was performed using the actin-encoding gene (*ACT1*) for normalization.

### Microscopy

For microscopy, the cells were routinely cultured in YPD medium overnight at room temperature (unless specified otherwise), diluted 1∶5 and incubated for an additional 4–5 hours. In some experiments, 10 µg/ml FK506 was added to the cultures 1 hour prior to microscopic observation. The cells were treated with 1 M NaCl or 100–300 mM CaCl_2_ for 5 min, pelleted and observed under the microscope within 30 min. CFW (1.5 mg/ml) was added to the cells for 1 hour followed by washing with YPD to remove debris. For heat shock, the cells were prepared for microscopy and the glass slides were placed on a heating block set on 42°C for 10 min. In some experiments, the cells were stained with 4′,6-diamidino-2-phenylindole (DAPI, 1 µg/ml) without fixation and immediately observed under the Deltavision Deconvolution microscope. Nuclear fluorescence was quantified using ImageJ software (NIH).

## Supporting Information

Figure S1 **Alignment of zinc finger-containing domains of fungal Crz1 proteins and two related transcription factors, ScAce2 and ScSwi5.** Cn_H99, Cryptococcus neoformans serotype A; Cn_JEC21, XP_566613, *C. neoformans* serotype D; An, BAE94327, *Aspergillus nidulans*; Ao, BAE57003, *Aspergillus oryzae*; Af, EAL88401, *Aspergillus fumigatus*; Mo, XP_359644, *Magnaporthe oryzae*; Nc, EAA32849, *Neurospora crassa*; EAQ88414, Chg, *Chaetomium globosum*; Gz, XP_381517, *Gibberella zeae*; Ci, XP_001244584, *Coccidioides immitis*; Pn, EAT87393, *Phaeosphaeria nodorum*; Dh, CAG84727, *Debaryomyces hansenii*; Td, AAZ04388, *Torulaspora delbrueckii*; Ag, AAS51722, *Ashbya gossypii*; Sc, CAA95889, *Saccharomyces cerevisiae*; Kl, CAG99429, *Kluyveromyces lactis*; Yl, CAG80473, *Yarrowia lipolytica*; Ca, EAK97605, *Candida albicans*; Cgl, CAG62620, *Candida glabrata*; Sp, Q09838, *Schizosaccharomyces pombe*; Cd, CAX43071, *Candida dubliniensis*; Sc_Ace2, CAA97702, *Saccharomyces cerevisiae*; Sc_Swi5, CAA90369, *Saccharomyces cerevisiae*. Identical amino acids: ‘*’, red. Strongly and weakly similar amino acids: ‘:’, green, and ‘.’, blue, respectively.
(PDF)

Figure S2 ***CRZ1*** **deletion,** *Δ* ***crz1*** **reconstitution, strategy to create** ***CRZ1-GFP*** **gene fusion, and verification of transformants.** (A) *CRZ1* promoter and coding region including introns were fused to *GFP*, terminator EF1T and neomycin resistance cassette in pCR2.1 cloning vector to create Δ*crz1* reconstitution construct (*EF1T*, elongation factor 1 terminator; *GFP,* green fluorescent protein; *NEO*, neomycin phosphotransferase; ActP, *ACT1* promoter; Ttrp, *TRP1* terminator) (B) To create GFP-tagged *CRZ1*, *GFP* was integrated downstream of the endogenous *CRZ1* by double cross-over recombination as indicated. Black arrows denote primers used in overlap PCR to create the construct and verify transformants. Primer regions not homologous to the template are indicated by diagonal lines. (C) Deletion of *CRZ1* by double cross-over recombination; (D and E) Verification of *CRZ1-GFP* expressing strain using genomic DNA and cDNA templates respectively; (F) Verification of *CRZ1*-*GFP* integration in Δ*cna1* mutant using genomic DNA as a template; (G) Confirmation of *CRZ1* gene deletion using genomic DNA as a template. Primers pairs used for transformant verification: 1. CRZ1-2462-s – a-GFP; 2. CRZ1-2462-s – GFP-a; 3. CRZ1-ots-s – GFP-a; 4. CRZ1-2462-s – CRZ1-3′UTR-a; 5. ActP-s – CRZ1.ExFP1; 6. Ttrp-s – CRZ1.ExRP2; 7. CRZ1-s – CRZ1-a. (Diagrams show shortened primer names).
(TIF)

Figure S3 **Virulence-related traits are not compromised in** Δ***crz1***. (A) Capsule size of WT H99, Δ*crz1* and Δ*cna1* was visualized following growth under capsule-inducing conditions. The G-protein α subunit mutant Δ*gpa1,* which has reduced capsule size, was used as a control for capsule induction. (B) Mating filaments production in crosses between WT KN99 MATa and WT, Δ*crz1* and Δ*cna1* (MATα), respectively. Consistent with Cruz, 2001, mating filament production was defective in the Δ*cna1* strain used here as a control (C) Melanization of the Δ*crz1* mutant as compared to WT and the melanin-deficient control strain, Δ*gpa1,* following growth on L-DOPA.
(TIF)

Figure S4 **Crz1-GFP fusion protein is functional as indicated by the transgenic strain resistance to calcofluor white.** Cells were spotted at 10-fold serial dilutions 10^6^–10 cells/spot from left to right.
(TIF)

Figure S5 **Continuously elevated temperature and heat shock cause different patterns of Crz1-GFP localization.** Being predominantly cytosolic at room temperature (A), Crz1-GFP translocates to the nuclei in cells grown at 37°C and 39°C (B, C), while abrupt temperature change (heat shock) causes punctate Crz1-GFP fluorescence concentrated in and around the nuclei (D).
(TIF)

Figure S6 **CFW fails to activate Crz1 in the calcineurin-deficient mutant.** Δ*cna1/*Crz1-GFP (A) and WT/Crz1-GFP (B) strains grown in YPD were exposed to CFW (1.5 mg/ml) for 1 hour. Nuclear targeting of Crz1-GFP in response to CFW is abolished in the Δ*cna1* mutant (C), while clearly detectable in WT (D).
(TIF)

Figure S7 **Crz1-GFP and polyA-binding protein Pab do not colocalize in salt-treated cells.** The heat maps represent fluorescence intensity of the nuclear stain (DAPI), Crz1-GFP and Pab-dsRed respectively. Black and white arrows map the exact location of some of the puncta where Crz1-GFP fluorescence is concentrated.
(TIF)

Table S1 **Primers used in this study.** The lowercase letters represent sequence with no homology to template DNA, whereas homologous regions are shown in uppercase.
(PDF)
