# Supplementary figures and images for: The Crz1/Sp1 Transcription Factor of Cryptococcus neoformans Is Activated by Calcineurin and Regulates Cell Wall Integrity
Source: PLoS One. 2012 Dec 12;7(12):e51403. doi: 10.1371/journal.pone.0051403 (PMC3520850; doi:10.1371/journal.pone.0051403)

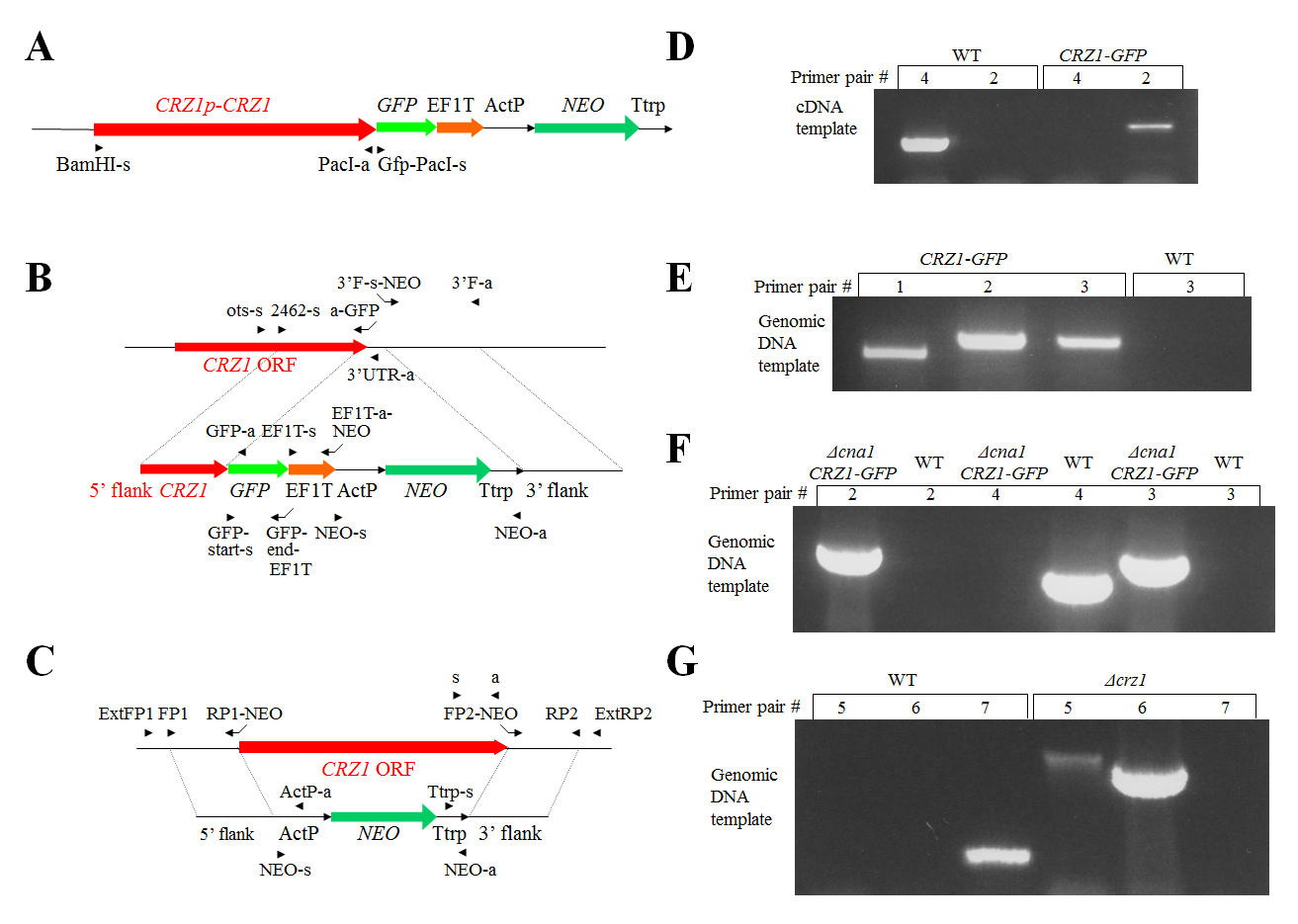

Supplement: Figure S2 — CRZ1 deletion, Δ crz1 reconstitution, strategy to create CRZ1-GFP gene fusion, and verification of transformants. (A) CRZ1 promoter and coding region including introns were fused to GFP, terminator EF1T and neomycin resistance cassette in pCR2.1 cloning vector to create Δcrz1 reconstitution construct (EF1T, elongation factor 1 terminator; GFP, green fluorescent protein; NEO, neomycin phosphotransferase; ActP, ACT1 promoter; Ttrp, TRP1 terminator) (B) To create GFP-tagged CRZ1, GFP was integrated downstream of the endogenous CRZ1 by double cross-over recombination as indicated. Black arrows denote primers used in overlap PCR to create the construct and verify transformants. Primer regions not homologous to the template are indicated by diagonal lines. (C) Deletion of CRZ1 by double cross-over recombination; (D and E) Verification of CRZ1-GFP expressing strain using genomic DNA and cDNA templates respectively; (F) Verification of CRZ1-GFP integration in Δcna1 mutant using genomic DNA as a template; (G) Confirmation of CRZ1 gene deletion using genomic DNA as a template. Primers pairs used for transformant verification: 1. CRZ1-2462-s – a-GFP; 2. CRZ1-2462-s – GFP-a; 3. CRZ1-ots-s – GFP-a; 4. CRZ1-2462-s – CRZ1-3′UTR-a; 5. ActP-s – CRZ1.ExFP1; 6. Ttrp-s – CRZ1.ExRP2; 7. CRZ1-s – CRZ1-a. (Diagrams show shortened primer names). (TIF) [file pone.0051403.s002.tif]

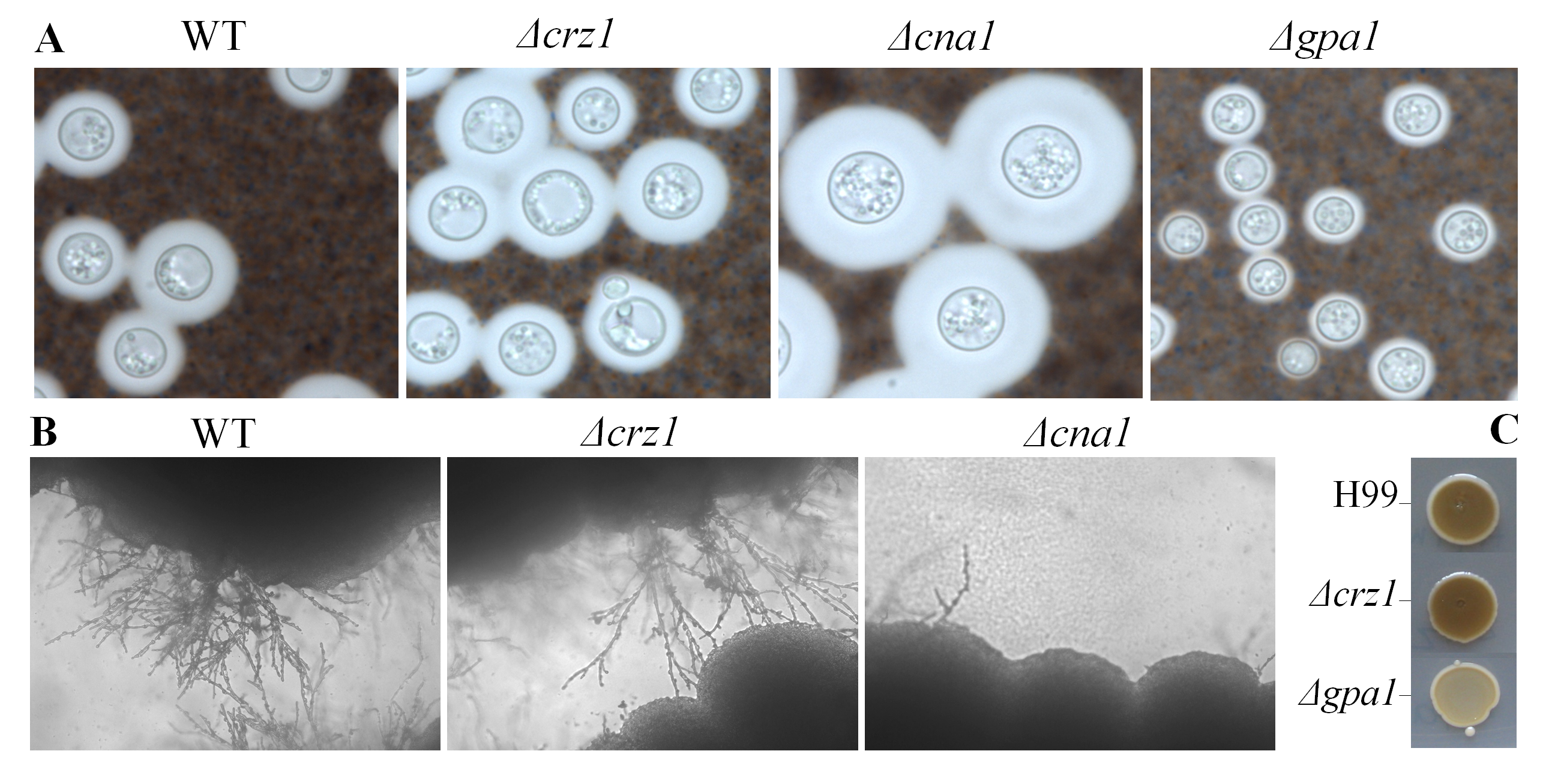

Supplement: Figure S3 — Virulence-related traits are not compromised in Δcrz1. (A) Capsule size of WT H99, Δcrz1 and Δcna1 was visualized following growth under capsule-inducing conditions. The G-protein α subunit mutant Δgpa1, which has reduced capsule size, was used as a control for capsule induction. (B) Mating filaments production in crosses between WT KN99 MATa and WT, Δcrz1 and Δcna1 (MATα), respectively. Consistent with Cruz, 2001, mating filament production was defective in the Δcna1 strain used here as a control (C) Melanization of the Δcrz1 mutant as compared to WT and the melanin-deficient control strain, Δgpa1, following growth on L-DOPA. (TIF) [file pone.0051403.s003.tif]

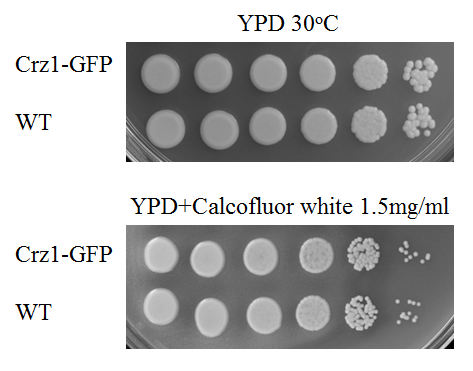

Supplement: Figure S4 — Crz1-GFP fusion protein is functional as indicated by the transgenic strain resistance to calcofluor white. Cells were spotted at 10-fold serial dilutions 106–10 cells/spot from left to right. (TIF) [file pone.0051403.s004.tif]

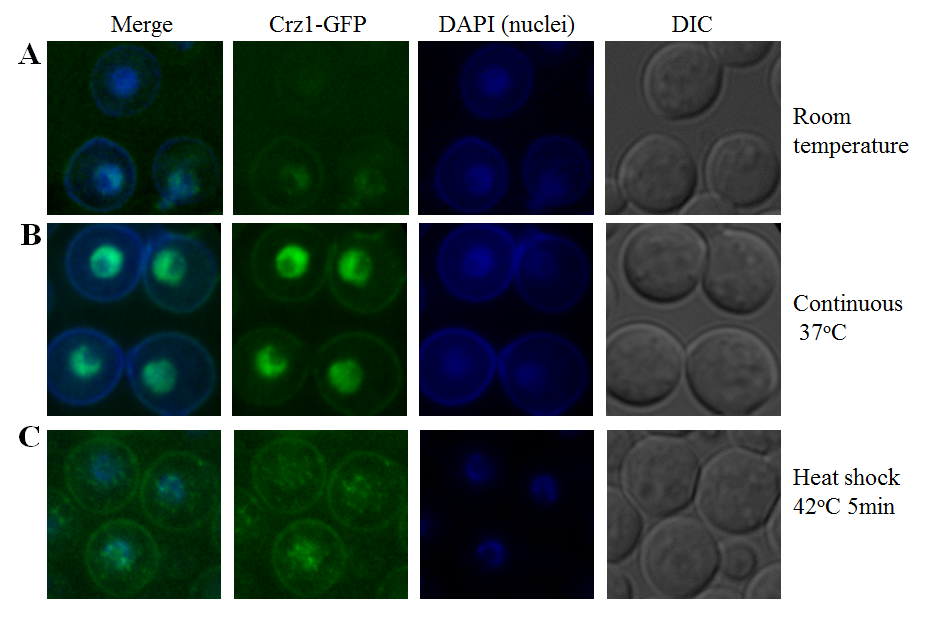

Supplement: Figure S5 — Continuously elevated temperature and heat shock cause different patterns of Crz1-GFP localization. Being predominantly cytosolic at room temperature (A), Crz1-GFP translocates to the nuclei in cells grown at 37°C and 39°C (B, C), while abrupt temperature change (heat shock) causes punctate Crz1-GFP fluorescence concentrated in and around the nuclei (D). (TIF) [file pone.0051403.s005.tif]

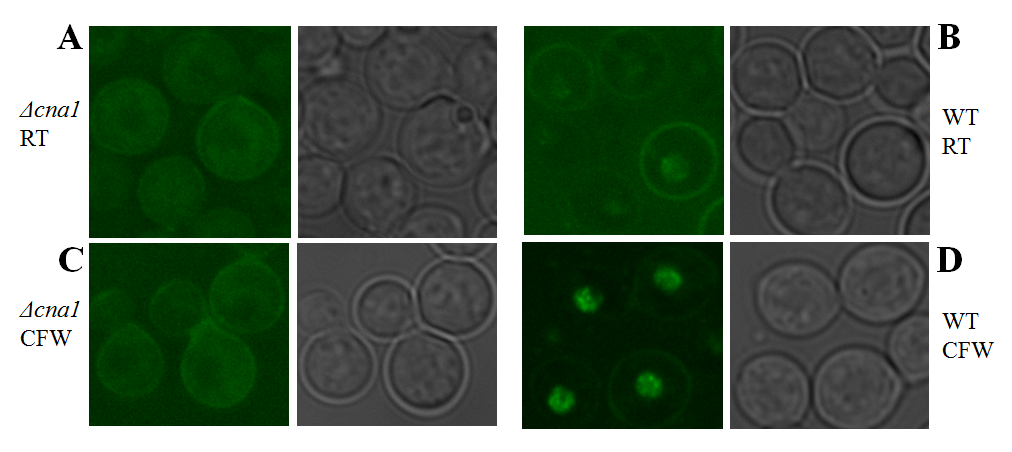

Supplement: Figure S6 — CFW fails to activate Crz1 in the calcineurin-deficient mutant. Δcna1/Crz1-GFP (A) and WT/Crz1-GFP (B) strains grown in YPD were exposed to CFW (1.5 mg/ml) for 1 hour. Nuclear targeting of Crz1-GFP in response to CFW is abolished in the Δcna1 mutant (C), while clearly detectable in WT (D). (TIF) [file pone.0051403.s006.tif]

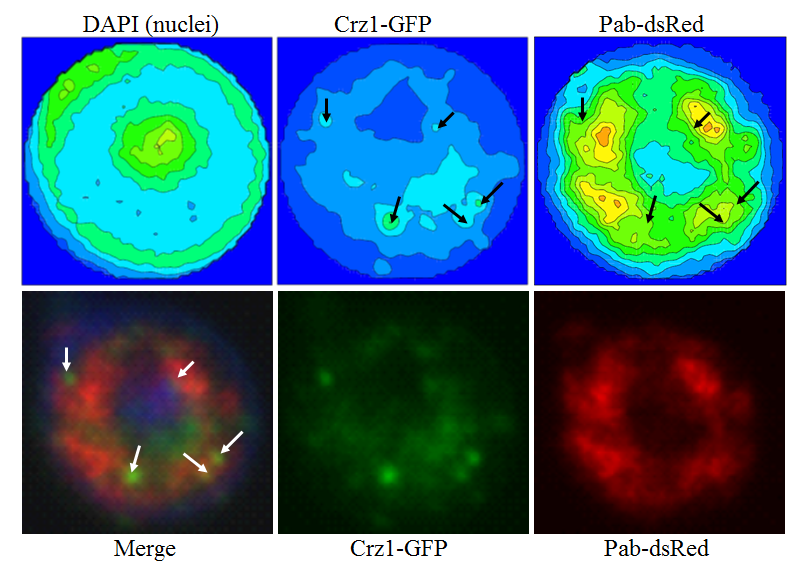

Supplement: Figure S7 — Crz1-GFP and polyA-binding protein Pab do not colocalize in salt-treated cells. The heat maps represent fluorescence intensity of the nuclear stain (DAPI), Crz1-GFP and Pab-dsRed respectively. Black and white arrows map the exact location of some of the puncta where Crz1-GFP fluorescence is concentrated. (TIF) [file pone.0051403.s007.tif]
