# Supplementary material for: The Crz1/Sp1 Transcription Factor of Cryptococcus neoformans Is Activated by Calcineurin and Regulates Cell Wall Integrity
Source: PLoS One. 2012 Dec 12;7(12):e51403. doi: 10.1371/journal.pone.0051403 (PMC3520850; doi:10.1371/journal.pone.0051403)
Supplement: Figure S1 — Alignment of zinc finger-containing domains of fungal Crz1 proteins and two related transcription factors, ScAce2 and ScSwi5. Cn_H99, Cryptococcus neoformans serotype A; Cn_JEC21, XP_566613, C. neoformans serotype D; An, BAE94327, Aspergillus nidulans; Ao, BAE57003, Aspergillus oryzae; Af, EAL88401, Aspergillus fumigatus; Mo, XP_359644, Magnaporthe oryzae; Nc, EAA32849, Neurospora crassa; EAQ88414, Chg, Chaetomium globosum; Gz, XP_381517, Gibberella zeae; Ci, XP_001244584, Coccidioides immitis; Pn, EAT87393, Phaeosphaeria nodorum; Dh, CAG84727, Debaryomyces hansenii; Td, AAZ04388, Torulaspora delbrueckii; Ag, AAS51722, Ashbya gossypii; Sc, CAA95889, Saccharomyces cerevisiae; Kl, CAG99429, Kluyveromyces lactis; Yl, CAG80473, Yarrowia lipolytica; Ca, EAK97605, Candida albicans; Cgl, CAG62620, Candida glabrata; Sp, Q09838, Schizosaccharomyces pombe; Cd, CAX43071, Candida dubliniensis; Sc_Ace2, CAA97702, Saccharomyces cerevisiae; Sc_Swi5, CAA90369, Saccharomyces cerevisiae. Identical amino acids: ‘*’, red. Strongly and weakly similar amino acids: ‘:’, green, and ‘.’, blue, respectively. (PDF) [file pone.0051403.s001.pdf]

```
                      10        20        30        40        50        60
                       |         |         |         |         |         |
Cn_H99       ASTSRRTNSGIFKCPVPGCGSTFTRHFNLKGHLRSHNDERPFKCLYEGCPKAIVGFARQHDCKR
Cn_JEC21     ASTSRRTNSGIFKCPVPGCGSTFTRHFNLKGHLRSHNDERPFKCLYEGCPKAIVGFARQHDCKR
An           --KRVQKHPATFQCTL--CPKRFTRAYNLRSHLRTHTDERPFVCT--VCGKA---FARQHDRKR
Ao           --KRVQKHPATFQCHL--CPKRFTRAYNLRSHLRTHTDERPFVCT--VCGKA---FARQHDRKR
Af           --KRVQKHPATFQCNL--CPKRFTRAYNLRSHLRTHTDERPFVCT--VCGKA---FARQHDRKR
Ci           --KRVQKHPATFQCNL--CPKRFTRAYNLRSHLRTHTDERPFVCT--VCGKA---FARQHDRKR
Chg          SAKRVQKHPATFQCTL--CPKRFTRAYNLRSHLRTHTDERPFVCT--VCGKA---FARQHDRKR
Mg           GPKRVQKHPATFQCSL--CPKRFTRAYNLRSHLRTHTDERPFVCT--VCGKA---FARQHDRKR
Gz           -TKRVQKHPATFQCTL--CPKRFTRAYNLRSHLRTHTDERPFVCT--VCGKA---FARQHDRKR
Nc           GPRRAQKHPATFQCKV--CPKRFTRAYNLRSHLRTHTDERPFKCT--VCDKA---FARQHDRKR
Pn           --KRTQKHPATFQCSL--CPKRFTRAYNLRSHLRTHTDERPFVCS--VCGKA---FARQHDRKR
Yl           ---RVQKHPSTFACHL--CDKRFTRSYNLRSHLRTHTDERPFVCT--VCGKA---FARQHDRKR
Td           -----QKNPAMFACEL--CDKRFTRPYNLKSHLRTHTNERPFICS--SCGKA---FARQHDRKR
Sc           --TNNRKNPANFACDV--CGKKFTRPYNLKSHLRTHTNERPFICS--ICGKA---FARQHDRKR
Cgl          -----NNTGTDYVCEV--CGKVFSRPYNLKSHLRTHTDEKPYQCS--ICGKA---FARQHDKKR
Kl           -----QKHPAVYACDI--CDKKFTRPYNLKSHLRSHTDERPYVCS--VCGKA---FARMHDKNR
Ag           ------KNPAIYSCNL--CDKKFTRPYNLKSHLRTHTDERPFSCS--VCGKA---FARQHDRKR
Ca           -SKRTQKHPSLYACHL--CDKRFTRPYNLKSHIRTHTQEKPFICS--KCGKS---FARSHDKKR
Cd           -SKRTQKHPSLYACHL--CDKRFTRPYNLKSHIRTHTQEKPFICS--KCGKS---FARSHDKKR
Dh           -SKRTQKHPSVYACHL--CDKRFTRPYNLKSHLRTHTDERPFICN--VCGKA---FARQHDRKR
Sp           KAKSESKSQGNYVCTFAGCNKRFTRAYNLKSHMNTHTNYRPFQCS--ICKKS---FARQHDKRR
Sc_Ace2      DQYVKELPDKLFECLYPNCNKVFKRRYNIRSHIQTHLQDRPYSCDFPGCTKA---FVRNHDLIR
Sc_Swi5      DKYVKEMPDKTFECLFPGCTKTFKRRYNIRSHIQTHLEDRPYSCDHPGCDKA---FVRNHDLIR
                        : *    * . *.* :*::.*:.:* : :*: *    * *:   *.* **  *

                      74        84        94       104       114       124
                       |         |         |         |         |         |
Cn_H99       HMLLHEGLRLFECEG----------CGKKFARLDALTRHHK---SEQGQECAITHPLPTNFDG
Cn_JEC21     HMLLHEGLRLFECEG----------CGKKFARLDALTRHHK---SEQGQECAITHPLPTNFDG
An           HEGLHSGEKKFVCRGDLSRGGQ-WGCGRRFARADALGRHFR---SEAGRICIKPLLDEESQER
Ao           HEGLHSGEKKFVCRGDLSRGGQ-WGCGRRFARADALGRHFR---SEAGRICIKPLLDEESQER
Af           HEGLHSGEKKFVCQGELSRGGQ-WGCGRRFARADALGRHFR---SEAGRICIKPLLDEESQER
Ci           HEGLHSGEKKFVCRGDLTRGGQ-WGCGRRFARADALGRHFR---SEAGRVCIKPLLDEEAAER
Chg          HEGLHSGEKKFVCKGELKRGGQ-WGCGRRFARADALGRHFR---SEAGRICIKPLLDEEISER
Mg           HEGLHSGEKKFVCKGDLKAGGQ-WGCGRRFARADALGRHFR---SEAGRICIKPLLDEEIMER
Gz           HESLHSGEKKFVCKGDLKVGGQ-WGCGRRFARADALGRHFR---SEAGRICIKPLLDEEMMER
Nc           HEGLHSGEKKFICKGELPVAGQQWGCGRRFARADALGRHFR---SEAGRICIRPLLEAENRER
Pn           HEGLHSGEKKFVCRGNLKDGNH-WGCGRRFARADALGRHFR---SEAGRVCIRPLLEEEAQEK
Yl           HEALHSGEKKYQCQGVLADGQTPWGCGRKFARADALGRHFK---TEAGRECIRLLLEEEANEK
Td           HEDLHTGKKRYVCGGHLKDGTA-WGCGKKFARSDALGRHFK---TESGRKCILPLYEEASREK
Sc           HEDLHTGKKRYVCGGKLKDGKP-WGCGKKFARSDALGRHFK---TESGRRCITPLYEEARQEK
Cgl          HEDLHTGKKRYVCGGKLKDGTF-WGCGKKFARSDALGRHFK---TSSGRKCITPLYEETARER
Kl           HEDLHTGKRRYVCGGILKNGNS-WGCGKKFARSDALGRHFK---TELGKKCILPLYEEAELEK
Ag           HEDLHSGKKRYVCGGKLKGGAT-WGCGKKFARSDALGRHFK---TESGRRCIAPLYDEASKER
Ca           HELLHQGIKNFKCEGYLQDGTR-WGCGKSFARADALRRHFQ---TEAGKQCVKRLLLEEQAN-
Cd           HELLHQGIKNFKCEGYLQDGTR-WGCGKSFARADALRRHFQ---TEAGKQCVKRLLLEEQAN-
Dh           HEDLHTGEKKFQCKGFLKDGTP-YGCGRKFARADALRRHFQ---TEAGKECIRLLVEEDEKDR
Sp           HEQLHTGIKAFACV----------TCNQRFARMDALNRHYK---SEVGQNCLRTATERGIQVP
Sc_Ace2      HKISHN-AKKYICP-----------CGKRFNREDALMVHRSRMICTGGKKLEHSINKKLTSPK
Sc_Swi5      HKKSHQ-EKAYACP-----------CGKKFNREDALVVHRSRMICSGGKKYENVVIKRSPRKR
             *   *   : : *            *.: * * ***  *        *:
```
