# Supplementary material for: The Crz1/Sp1 Transcription Factor of Cryptococcus neoformans Is Activated by Calcineurin and Regulates Cell Wall Integrity
Source: PLoS One. 2012 Dec 12;7(12):e51403. doi: 10.1371/journal.pone.0051403 (PMC3520850; doi:10.1371/journal.pone.0051403)
Supplement: Table S1 — Primers used in this study. The lowercase letters represent sequence with no homology to template DNA, whereas homologous regions are shown in uppercase. (PDF) [file pone.0051403.s008.pdf]

Table S1. Primers used in this study. The lowercase letters represent sequence with no homology to template DNA, whereas homologous regions are shown in uppercase.

CRZ1-GFP construct and transformant confirmation				
Neomycin and Nourseotricin resistance cassettes	NEO-s	CTGCGAGGATGTGAGCTGGAG	NEO-a	GGAGCCATGAAGATCCTGAGG
GFP coding sequence	GFP-start-s	TCCAAGGGTGAGGAGCTCTTC	GFP-end-a-EF1T	agaatgggctctacagaagcgaTTAGAGGTCCTCCTCGGAGATGAG
EF1 terminator	EF1T-s	TCGCTTCTGTAGAGCCCATTCT	EF1T-a-NEO	ctccagctcacatcctcgcagGACACCTTCACCTTGATCTCTTCC
5’ flank	CRZ1-2462-s	GAGTGGGAGCTGGTGGTTCAAC	CRZ1-a-GFP	gaagagctcctcacccttggaATCCTCTTCACTCGTTTCACTCTTC
3’ flank	CRZ1-3'F-s-NEO	cctcaggatcttcatggctccCCGATGGTCATAGGGCGCTGT	CRZ1-3'F-a	GGTATGAGGCGACCTAAGGGT
Transformant confirmation	CRZ1-ots-s	GGAAGAGGTGGTACAGGATGG	GFP-a	GGTCTTGTAGTTACCGTCGTCC
Transformant confirmation - CRZ1 expression			CRZ1-3'UTR-a	TTCAGCTCACAGCGCCCTATG
CRZ1 deletion construct and mutant confirmation				
5’ flank	CRZ1.FP1	TGGTTCGTTAGTCGGGTCAA	CRZ1.RP1-NEO	tccagctcacatcctcgcagAGGCTGGATCTGCCATTTTT
3’ flank	CRZ1.FP2-NEO	cctcaggatcttcatggctccAAAAGGCGGTCGAAGAAGAG	CRZ1.RP2	CTAGATGATGAGGGCGAACG
Mutant confirmation	CRZ1.ExtFP	GTGTGAGTGAAGGCGGAGAG	CRZ1.ExtRP	AAGAGGCCAAAGGAGATTGC
Neo cassette internal primers	Ttrp-s	CTACAGACAACAATACCATCCTTCC	ActP-a	TGTTGTTACCATCATCCTCTCCTC
CRZ1 internal primers	CRZ1-s	TGTCCCAAAGCTATCGTCGGT	CRZ1-a	ATCCTCTTCACTCGTTTCACTCTTC
Pab-dsRed construct and transformant confirmation				
5’ flank	Pab-5'-s	GGTTATGAGGGACGACAGTGGTG	Pab-5'-a-Red	atgacgtcctcggaggaggcCTCCTTGGTCTCCTCCTCTTCC
3’ flank	Pab-3'-s-NEO	cctcaggatcttcatggctccCCATTCTTATCCATCCTATCCACC	Pab-3'-a	GTTCTTCAGCGAGCATGGCAA
dsRed coding sequence	Red-s	GCCTCCTCCGAGGACGTCAT	Red-a-EF1T	agaatgggctctacagaagcgaCTACAGGAACAGGTGGTGGCG
Transformant confirmation	Pab-5'-ots-s	AGATGAGTGGGATGACGACCG	Pab-3'-ots-a	CTCATCATCGTAAACCAGCCCAT
CHS6 expression - sqPCR				
CHS6	CHS6-s	TCATCAGAGGAAGCAAGCCACA	CHS6-2	CTTCAACTTATCCAGCACCTCCTC
ACT1	ACT-s	GCCCAGTCTTCTCAGCTTGAAA	ACT1-a	ACTTTCGGTGGACGATTGAGG
Yeast two hybrid				

CRZ1	CRZ1-XmaI-s	aattcccgggatTGGCAGATCCAGCCTCACCC	CRZ1-XhoI-a	acatctcgagTTAATCCTCTTCACTCGTTTCACTC
CNA1	CNA1-EcoRI-s	ctaagaattcGCTTCCCCAGCCACTCAGAC	CNA1-BamHI-a	taatggatccTCACTCGCCTTGACCGCCCT
